# Sperm function is required for suppressing locomotor activity of *C*. *elegans* hermaphrodites

**DOI:** 10.1371/journal.pone.0297802

**Published:** 2024-01-25

**Authors:** Satoshi Suo

**Affiliations:** Department of Pharmacology, Faculty of Medicine, Saitama Medical University, Saitama, Japan; Baylor University, UNITED STATES

## Abstract

Sex differences in sex-shared behavior are common across various species. During mating, males transfer sperm and seminal fluid to females, which can affect female behavior. Sperm can be stored in the female reproductive tract for extended periods of time and used to fertilize eggs. However, the role of either sperm or embryo production in regulating female behavior is poorly understood. In the androdioecious nematode *C*. *elegans*, hermaphrodites produce both oocytes and sperm, enabling them to self-fertilize or mate with males. Hermaphrodites exhibit less locomotor activity compared to males, indicating sex difference in behavioral regulation. In this study, mutants defective in the sperm production and function were examined to investigate the role of sperm function in the regulation of locomotor behavior. Infertile hermaphrodites exhibited increased locomotor activity, which was suppressed after mating with fertile males. The results suggest that sperm, seminal fluid, or the presence of embryos are detected by hermaphrodites, leading to a reduction in locomotor activity. Additionally, females of closely related gonochoristic species, *C*. *remanei* and *C*. *brenneri*, exhibited reduced locomotor activity after mating. The regulation of locomotion by sperm function may be an adaptive mechanism that enables hermaphrodites lacking sperm or embryo to search for mates and allow females to cease their search for mates after mating.

## Introduction

There are sex differences in behavior, including mating behavior and shared behaviors such as locomotion [1–3]. Both internal and external stimuli are known to regulate these behaviors. Mating can influence the behavior of females, particularly in insects [4, 5]. Males transfer sperm and seminal fluid to the female body through mating. Previous studies have indicated that seminal proteins may regulate the behavior of female flies, resulting in increased aggression and decreased receptivity to males after mating. Sperm can be stored in the female reproductive tract and these storages are called sperm reservoir. In wide-ranging animal species, including vertebrates and invertebrates, sperm reservoir has been shown to persist for extended periods of time [6, 7]. However, the specific effects of insemination or embryo production on female behavior remain largely unknown.

*C*. *elegans* has two sexes: male and hermaphrodite [8]. *C*. *elegans* hermaphrodites are morphologically female, but produce both oocytes and sperm, allowing them to self-fertilize and also mate with males. Hermaphrodites produce sperm during their larval stage, store them in the spermatheca, and transition to producing eggs upon reaching adulthood. When males mate with hermaphrodites, their sperm crawl to the spermatheca and at least partially displaces the hermaphrodite-derived sperm [9]. During fertilization, the male-derived sperm is preferred over hermaphrodite-derived sperm. Therefore, most of the progenies are born from cross-fertilization after mating. It remains largely unknown how the presence of sperm or embryos affects the behavior of hermaphrodites.

*C*. *elegans* exhibits two distinct behavioral states, dwelling and roaming [10]. Dwelling animals move slowly and frequently change direction, whereas roaming animals exhibit higher locomotor activity and turn less frequently. There are sex differences in the regulation of these behavioral states, whereby hermaphrodites spend more time in the dwelling state, while males spend more time in the roaming state [11]. This allows males, which require mates to reproduce, to explore large areas to find hermaphrodites.

*C*. *elegans* hermaphrodites possessing self-sperm provide an opportunity to investigate the effect of stored sperm using sperm-defective mutants without confounding effects of mating. In the present study, I examined the locomotion of mutants defective in sperm function to test whether sperm function influences the regulation of behavioral states. The results showed that sperm-defective hermaphrodites display elevated locomotor activity and increased roaming behavior, suggesting that the sperm function plays a role in regulating sex-shared behavior in *C*. *elegans*, potentially through the production of fertilized eggs. This study sheds new light on the potential contribution of internal sperm to the regulation of behavior.

## Materials and methods

### Strains

Culturing of *C*. *elegans* was performed as previously described [8]. The strains used in this study are as follows: N2 wildtype (RRID:WB-STRAIN:N2), CB4088 *him-5(e1490)* (RRID:WB-STRAIN:CB4088), BA821 *spe-26(hc138)* (RRID:WB-STRAIN:BA821), BA-17 *fem-1(hc17)* (RRID:WB-STRAIN:BA17), CB4108 *fog-2(q71)* (RRID:WB-STRAIN:CB4108), CB3844 *fem-3(e2006)* (RRID:WB-STRAIN:CB3844), BA671 *spe-9(hc88)* (RRID:WB-STRAIN:BA671), EM464 *C*. *remanei* (RRID:WB-STRAIN:EM464), and CB5161 *C*. *brenneri* (RRID:WB-STRAIN:CB5161). CB4088 *him-5(e1490)* was used to obtain males for mating with *C*. *elegans* strains.

### Image acquisition and data analysis

The analyses of *C*. *elegans* locomotor behavior were performed as previously described [11] with some modifications. The assay plates were made of low peptone-NGM agar (0.25 g/L peptone, 3 g/L NaCl, 17 g/L Agar, 25 mM KPO4 (pH 6.0), and 5 mM MgSO4, 5 mM CaCl2). 10 μL of OP50 (RRID:WB-STRAIN:OP50) suspension was placed on the plates to create thin and small bacterial lawns.

Animals were grown at 16°C. For the experiments with N2 and *spe-26*, L4 animals were picked from the 16°C plates and placed onto a 40 mm NGM plate [8] seeded with OP50 and were grown at 22°C for approximately 20 h. In this condition, *spe-26* animals produced no viable progenies and only laid unfertilized eggs. Varkey et al. previously showed that *spe-26(hc138)* mutants were completely infertile at 25°C [12]. Therefore, it remains possible that these animals were hypomorphic at 22°C, even though they produced no progeny. For the experiments with *fem-1*, *fem-3*, *fog-2* and *spe-9*, adult animals were transferred from the 16°C and grown at 22°C for 2 days. Then L4 animals were pick to new plates and grown at 22°C for approximately 20 h. With this condition, *fog-2* produced no viable progenies whereas *fem-1*, *fem-3* and *spe-9* produced some progenies.

In the mating condition, L4 hermaphrodites (or females) were cultured together with L4 males of the same species for 20 h at 22°C. Subsequently, the animals were individually transferred to the assay plates 15 min prior to recording. The images of the assay plates were captured using a DMK series USB camera (Imaging Source, Bremen, Germany) with a resolution of 2,000 x 1,944 pixels, at one frame per sec for 25 min, using the gstreamer software with Raspberry Pi 3 (Raspberry Pi Foundation, Cambridge, United Kingdom).

For the measurements with L4 animals, L4 animals of N2 and *spe-26* were picked from plates kept at 22°C for 2 days and transferred to the assay plates.

The position of the animals within the bacterial lawn was obtained using a custom-written code in Python and the average speed and angular speed were determined. To analyze behavioral states, the average speed and angular speed of 10-sec intervals were determined and plotted [11]. Data points located above a predefined line (Speed (μm/s) = 2 x Angular Speed (degree/s) + 100) were classified as roaming and those below were categorized as dwelling, similarly as described in a previously study [13]. In our previous study [11], data points from males and hermaphrodites were categorized through a cluster analysis. The categorization using this line was consistent with the cluster analysis and 99.5% of data points resulted in the same categorization in both N2 and CB1112 *cat-2* animals.

### Sperm staining

Males were stained with MitoTracker Red CMXRos (ThermoFisher, Waltham, MA) as previously described [14]. L4 males were picked onto MitoTracker containing plates, prepared by placing 100 μL of 10 μM Mitotracker on OP50-seeded NGM plates. After growing 16 h at 20°C, adult males were transferred to new plates without Mitotracker and mated with adult hermaphrodites (or females) for 6 h. The hermaphrodites were then mounted on slides and their images were taken using a fluorescence microscope (BX53, Olympus, Tokyo, Japan).

### Statistical analysis

Numbers of animals tested for each experiment are shown in the figure legends. Statistical analysis was performed using Python. The Wilcoxon rank-sum test was used to determine the p values. The Bonferroni correction was used for comparisons of more than two groups. For comparisons of behavioral states, the percent of time spent in roaming state was compared between strains and conditions. The differences were considered statistically significant with *p < 0.05, **p < 0.01, ***p < 0.001. For the box plots, the boxes depict the lower and upper quartile values, and the central lines represent the medians. The whiskers are the most extreme values within 1.5 times the interquartile range and the dots represent outliers.

## Results

### Sperm-defective animals exhibit increased locomotor activity

**Fig 1:**
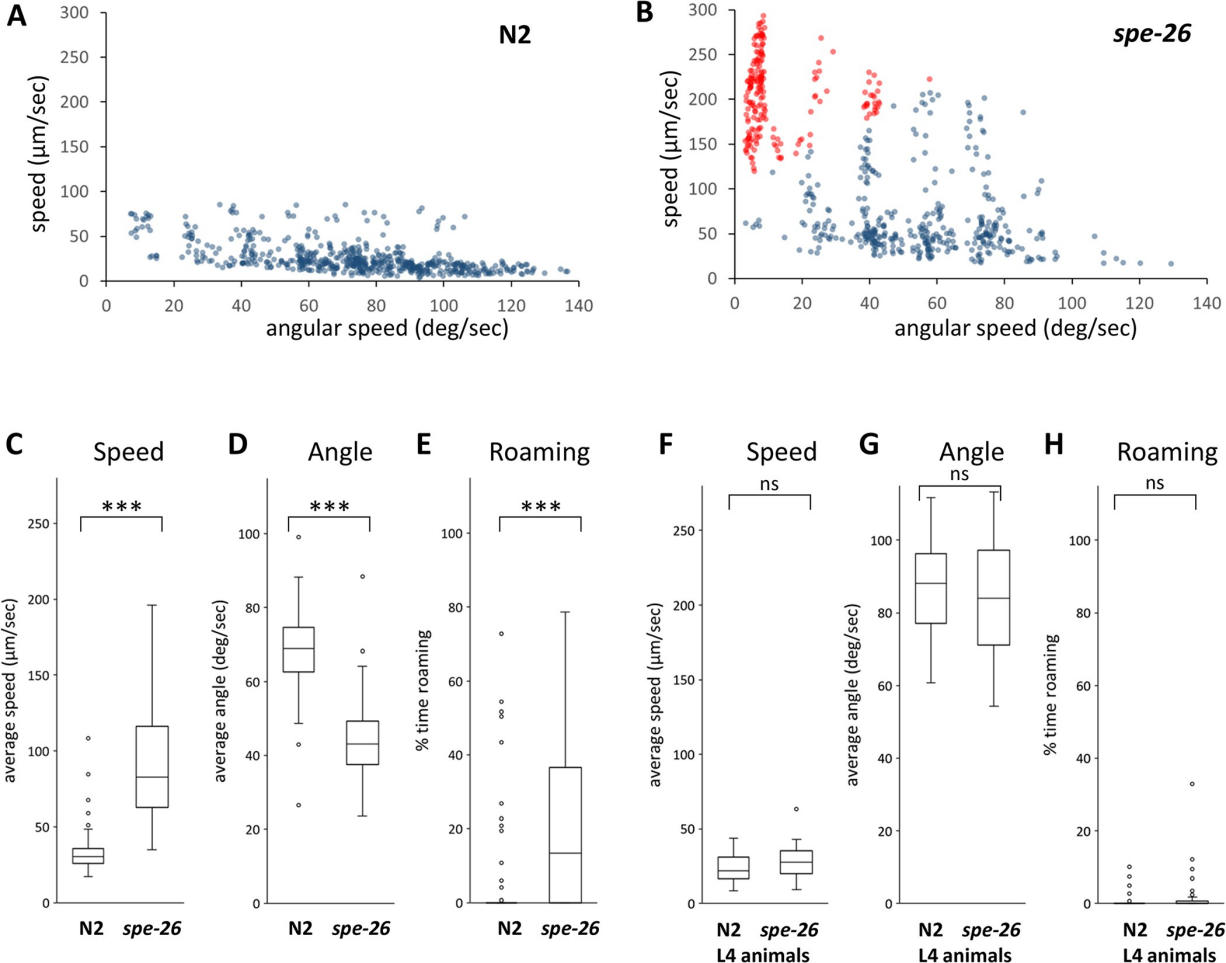
Locomotor behavior of N2 and *spe-26* hermaphrodites. Scatter plots of average speed and average angular speed in 10-sec intervals for 16 each N2 adult hermaphrodites (A) and sperm-defective *spe-26* mutant adult hermaphrodites (B). Data points were categorized into roaming (red) and dwelling (blue). Average speed (C), angular speed (D), and the percentage of time spent in the roaming state (E) were determined. L4 animals of N2 and *spe-26* mutants were also examined (F-H). Numbers of the animals tested: N2, 75; *spe-26*, 79; L4 N2, 44; L4 *spe-26*, 38.

In this study, I investigated the locomotor behavior of *C*. *elegans* animals cultured on plates containing food bacteria. We recorded the behavior of individually placed animals and determined the average speed and average angular speed of 10-second intervals. Consistent with previous studies [11, 13, 15], wildtype hermaphrodite animals exhibited low speed and high angular speed. High angular speed indicates frequent turning of animals. Data points were plotted (Fig 1A), with many points located in the low-speed and high-angle region, and fewer points located in the high-speed and low-angle region. Similar to a previous study [13], we separated the behavioral states. Points with high speed and low angular speed were categorized as the roaming state, while points with low speed and high angular speed were categorized as the dwelling state. Our findings confirmed that wildtype animals spent most of their time in the dwelling state.

*spe-26* mutants are spermatogenesis-defective and their spermatocytes arrest without dividing into functional sperm [12]. Hermaphrodites of the *spe-26* mutant were tested to investigate the effect of sperm on locomotor behavior. We recorded and plotted the data points for the *spe-26* hermaphrodites and found that *spe-26* hermaphrodites exhibited a greater number of points with high-speed and low-angle, when compared to their wildtype counterparts (Fig 1B). The *spe-26* hermaphrodites exhibited increased speed, decreased angular speed, and increased time spent in the roaming state, when compared to wildtype hermaphrodites (Fig 1C–1E). Furthermore, these behavioral differences were not observed when wildtype and *spe-26* mutant animals were tested at the L4 stage in which sperm is not fully developed (Fig 1F–1H). These results suggest that functional sperm suppresses roaming behavior and the loss of sperm results in an increase in locomotor activity.

**Fig 2:**
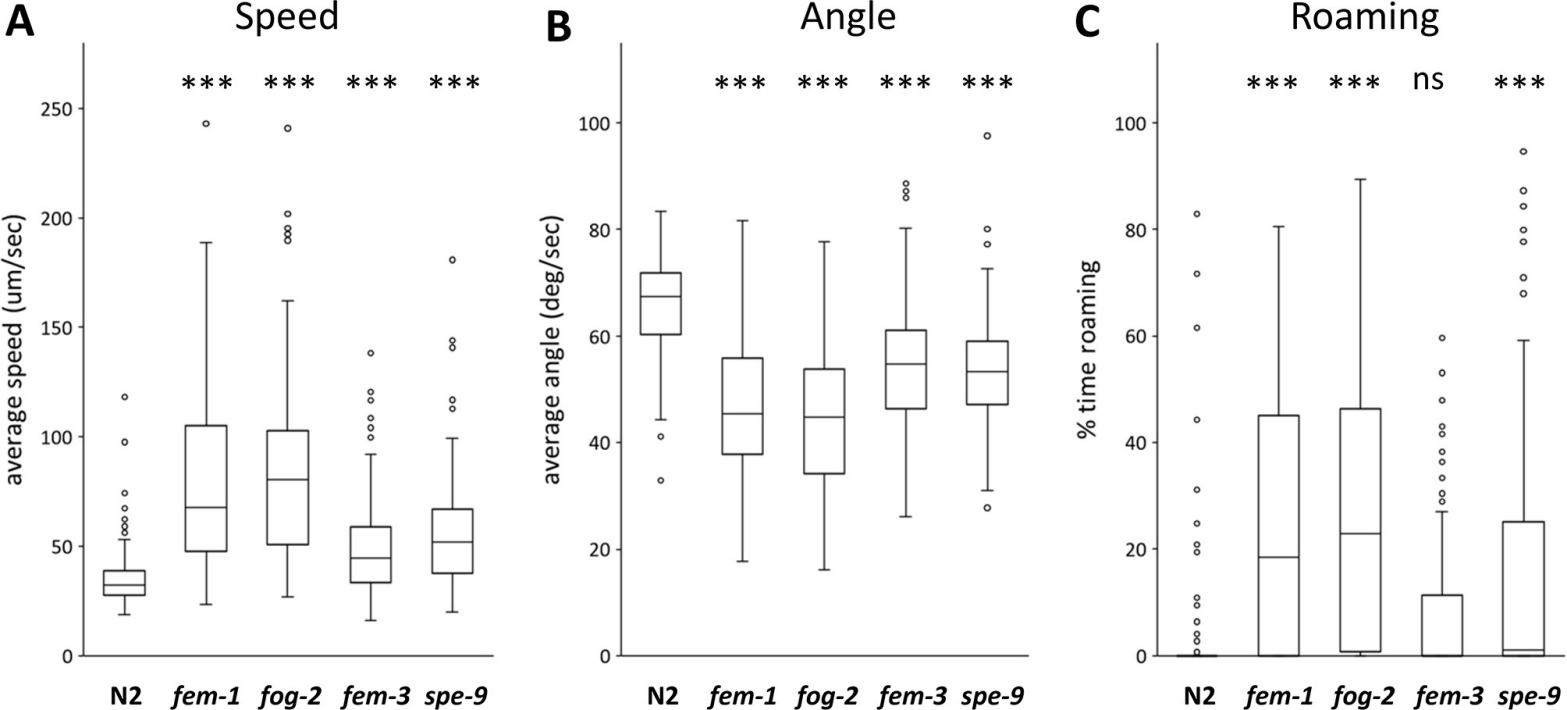
Locomotor behavior of sperm-defective mutants. Average speed (A), angular speed (B), and the percentage of time spent in the roaming state (C) of the *fem-1*, *fog-2*, *fem-3*, and *spe-9* mutants were determined. Numbers of the animals tested: N2, 91; *fem-1*, 93; *fog-2*, 91; *fem-3*, 92; *spe-9*, 92.

In addition to the *spe-26* mutants, I also examined other sperm-defective mutants (Fig 2). The *fem-1*, *fem-3*, and *fog-2* genes work in the sex-determination pathway; mutant hermaphrodites are “feminized” since they produce only oocytes [16–18]. *spe-9* mutants are spermatogenesis-defective mutants that make motile sperm that can reside in the spermatheca and stimulate oocyte maturation and ovulation, but cannot fertilize oocytes [19]. I examined the locomotor behavior of the hermaphrodites of these mutants and found that all of them had significantly increased speed and decreased angular speed compared to wildtype hermaphrodites. Furthermore, the roaming behavior was also increased in *fem-1*, *fog-2*, and *spe-9* mutants. In this experimental condition (22°C), *spe-26* and *fog-2* could be hypomorphic even though they produced no viable progenies. *fem-1*, *fem-3* and *spe-9* produced some progenies, suggesting that these mutants were not complete loss-of-function. As several mutants that cause defect in sperm function in different manners each showed increased locomotor activity, the results further support the notion that sperm function is required for suppression of locomotion in hermaphrodites. Given that *spe-9* hermaphrodites, which possess largely normal sperm functions except for the ability to fertilize eggs, exhibited increased activity, it is possible that the production of fertilized eggs, rather than sperm itself, alters hermaphrodite locomotion.

### Mating suppresses increased locomotion of sperm-defective animals

**Fig 3:**
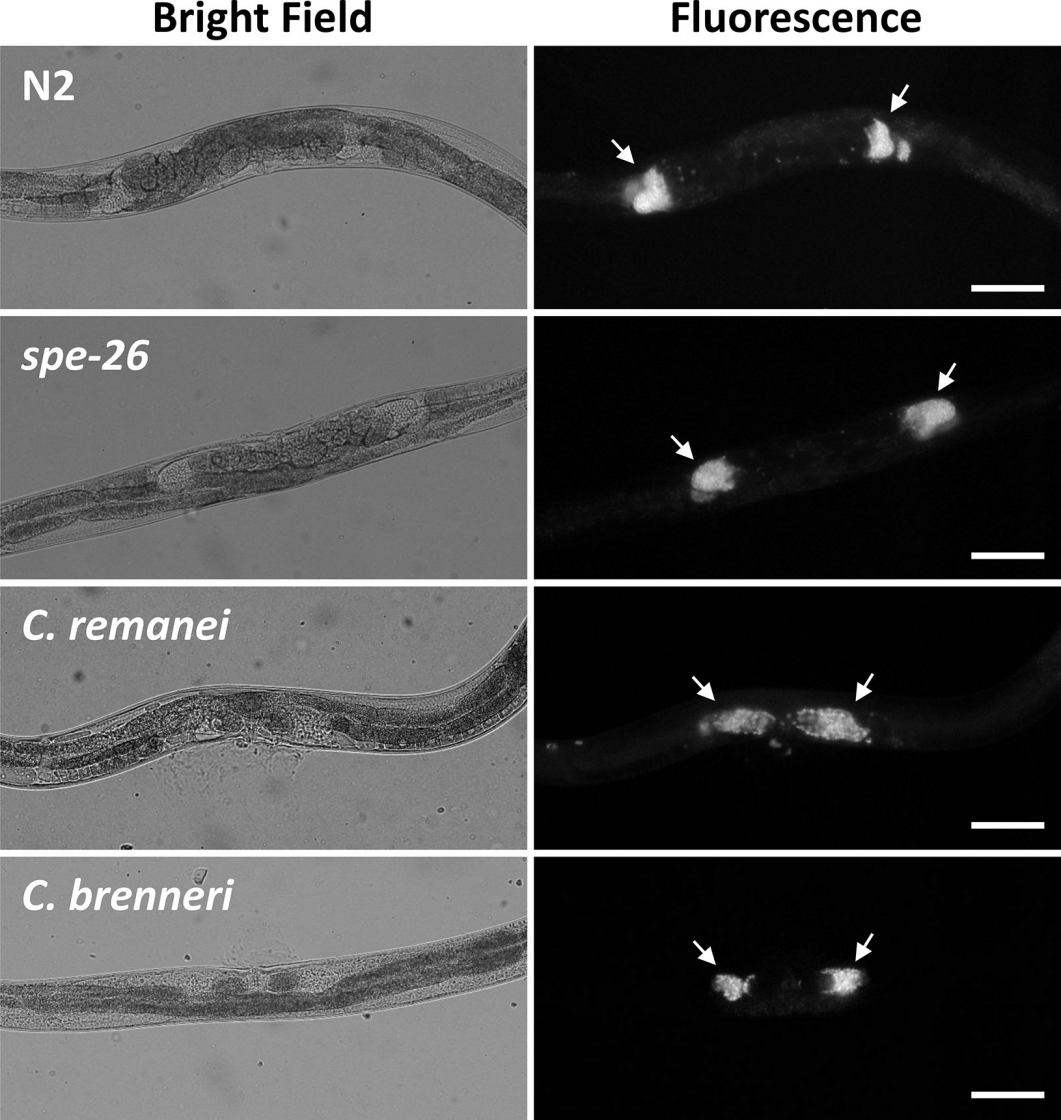
Transfer of sperm. N2 and *spe-26 C*. *elegans* hermaphrodites were mated with CB4088 *him-5* males stained with MitoTracker and *C*. *remanei* and *C*. *brenneri* females were mated with corresponding males stained with MitoTracker, and the bright field and fluorescence images were obtained. Arrows indicate possible sperm fluorescence accumulating at the location of spermatheca. Scale bar = 10 μm.

**Fig 4:**
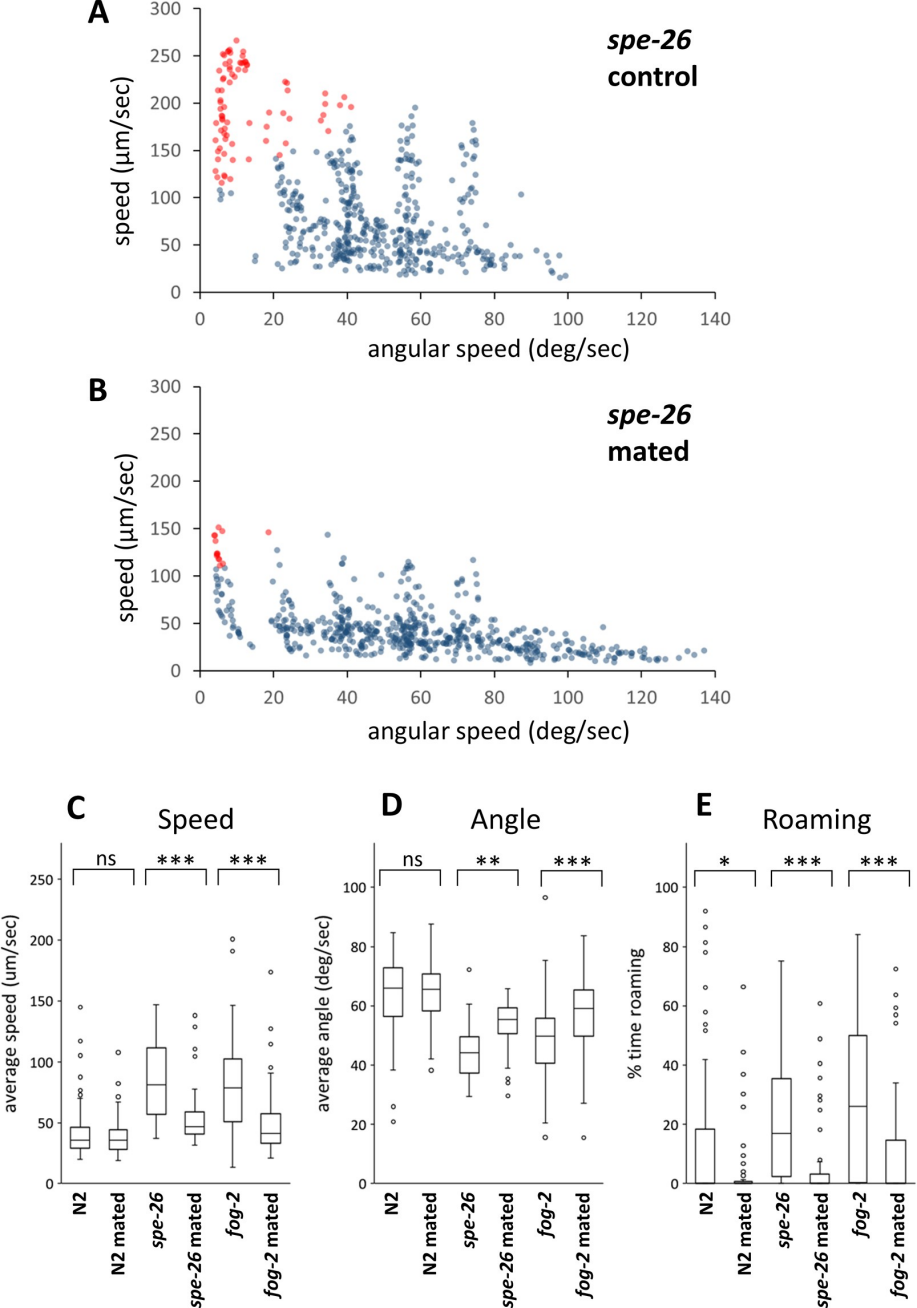
Locomotor behavior of N2 and *spe-26* hermaphrodites after mating. Scatter plots of average speed and average angular speed in 10-sec intervals for 16 each of control *spe-26* hermaphrodites (A) and *spe-26* hermaphrodites mated with males (B). Data points were categorized into roaming (red) and dwelling (blue). Average speed (C), angular speed (D), and the percentage of time spent in the roaming state (E) were determined for N2, *sper-26* and *fog-2* hermaphrodites with or without mating. Numbers of the animals tested: N2 control, 77; N2 mated, 75; *spe-26* control, 61; *spe-26* mated, 64; *fog-2* control, 60; *fog-2* mated, 63.

The effect of adding sperm to hermaphrodites on locomotion was investigated to determine whether sperm can suppress increased locomotion in sperm-defective mutants. Male-derived sperm moves to the spermatheca after mating and can be stored for an extended period [9]. To confirm the presence of male-derived sperm in hermaphrodites, MitoTracker-stained *him-5* males were mated with wildtype and *spe-26* hermaphrodites, and fluorescent cells, most likely sperm, were observed in both wildtype and *spe-26* hermaphrodites (Fig 3). For the behavioral analysis, the movements were recorded from both wildtype and *spe-26* hermaphrodites that had either mated or not mated with *him-5* males. There were less data points with high speed and low angular speed in mated *spe-26* mutants compared to unmated *spe-26* mutants (Fig 4A and 4B). *spe-26* mutants exhibited increased speed, decreased angle, and increased roaming compared to wildtype hermaphrodites. However, these locomotor changes were reduced by mating (Fig 4C–4E). The similar results were also obtained when *fog-2* mutants were tested (Fig 4C–4E). In contrast, in wildtype hermaphrodites, mating did not significantly alter their speed and angle, although roaming was reduced. These results demonstrate that mating suppresses the increased locomotion of sperm-defective animals.

### Mating suppresses locomotion in females of other *Caenorhabditis* species

**Fig 5:**
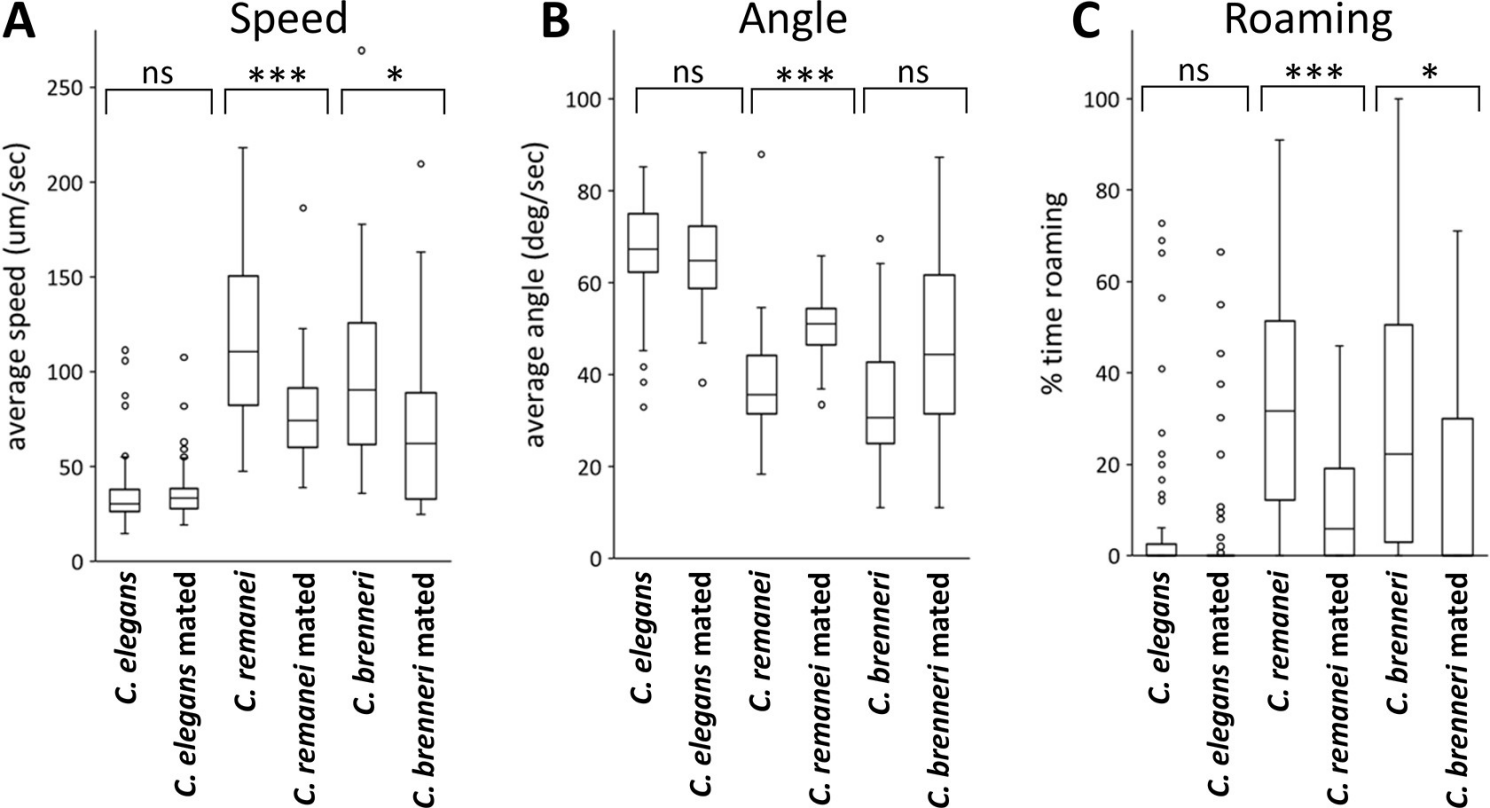
Locomotor behavior of *Caenorhabditis* species after mating. Average speed (A), angular speed (B), and the percentage of time spent in the roaming state (C) of *C*. *elegans* hermaphrodites, *C*. *remanei* females, and *C*. *brenneri* females were determined. For mated, animals were mated with corresponding males prior to recording. The average speed of unmated animals was higher in *C*. *remanei* (p < 0.01) and *C*. *brenneri* (p < 0.01), when compared to *C*. *elegans*. Numbers of the animals tested: *C*. *elegans* control, 76; *C*. *elegans* mated, 76; *C*. *remanei* control, 61; *C*. *remanei* mated, 74; *C*. *brenneri* control, 39; *C*. *brenneri* mated, 46.

*C*. *elegans* is an androdioecious species, which has males and hermaphrodites, whereas other closely related species, such as *C*. *remanei* and *C*. *brenneri*, are gonochoristic and have male and female sexes. I also investigated whether mating regulates locomotion in these gonochoristic species. First, dye-stained males were mated with females, and it was confirmed that male-derived sperm is stored in the female reproductive tracts (Fig 3). Behavioral analyses revealed that females of these gonochoristic species exhibit a higher locomotor rate than *C*. *elegans* hermaphrodites. Furthermore, there were a decrease in speed, an increase in angle, and an increase in roaming after mating with males in *C*. *remanei* and *C*. *brenneri* females, similar to what was observed in the sperm-defective *C*. *elegans* mutants (Fig 5). These results suggest that the behavior of female nematodes changes after mating, possibly due to sperm transfer.

## Discussion

This study demonstrated that the hermaphrodites of mutants with defective sperm function exhibit increased locomotor activity compared to wildtype hermaphrodites. The consistent increase in locomotor activity across multiple mutants with defects in sperm development or function supports the idea that sperm plays a role in suppressing locomotor activity in hermaphrodites. The *fem-1*, *fem-3*, and *fog-2* mutants have defects in germ-line sex determination and produce oocytes instead of sperm [16–18]. The *spe-26* mutants have a defect in the early stages of sperm development and produce aberrant spermatocytes that do not progress to spermatids [12]. In contrast, *spe-9* mutants produce spermatozoa with wild-type morphology, but these sperm are not functional and cannot fertilize oocytes [19]. These results suggest that the presence of mature functional sperm and the resulting production of embryos is necessary for suppressing locomotor activity in adult hermaphrodites.

The effect of mating on hermaphrodite’s behavior was also examined. The results showed that sperm-defective *spe-26* hermaphrodites exhibit a reduction in locomotor activity after mating with wildtype males, which results in both the transfer of male-derived sperm and the production of viable embryo. This further supports the idea that the presence of functional sperm suppresses locomotor activity in *C*. *elegans*. However, these findings do not rule out the possibility that other factors also play a role in regulating locomotor activity. Mating can trigger modifications in behavior and physiology through the transfer of seminal fluid [4, 5, 20]. In addition, the chemical and physical perception of males may also play a role in this process. Therefore, it is possible that the observed decrease in locomotor activity in this study is not solely mediated by sperm or embryo production but could be a result of combination of various factors.

In our previous study, we observed that *C*. *elegans* males exhibit higher levels of locomotor activity and spend more time in the roaming state than hermaphrodites [11]. This sex difference in locomotion may be adaptive, as males require mates to reproduce and therefore benefit from exploring larger areas to find potential mates. In contrast, hermaphrodites are capable of self-fertilization and may conserve energy by remaining in areas with ample food. The present study showed that the loss of sperm function results in increased locomotor activity of hermaphrodites. Sperm-defective hermaphrodites, which effectively are females, require males to reproduce. Therefore, this behavioral change may be adaptive, as it makes the sperm-defective hermaphrodites search a larger area for mates.

There are male-female species of nematodes closely related to *C*. *elegans*. Notably, the females of two such strains, *C*. *remanei* and *C*. *brenneri*, were more active than *C*. *elegans* hermaphrodites. Furthermore, a decrease in locomotor activity and roaming behavior was observed in mated females, who received sperm from males. It remains possible that the locomotor activity of females is not regulated by sperm itself but other mating associated factors, including copulatory plugs left by males around female vulva, which might slow down the movement, and the production of embryos. Nonetheless, the mating-induced behavioral regulation would allow females to reduce locomotion and remain near available food sources after mating, when they no longer need to search for mates.

Sperm status affects global gene expression in *C*. *elegans* [21] and alters aspects of hermaphrodite physiology in *C*. *elegans*. Furthermore, sperm facilitates the maturation of oocytes [22], increases cold tolerance [23], protect from mating-induced death [24, 25] and reduces attractiveness to males [26]. This study expands on these findings and demonstrate that sperm plays a role in regulating locomotor behavior. The mechanism by which sperm regulates hermaphrodite behavior is currently unknown. One possibility is that sperm itself or factors released from sperm is detected by hermaphrodite cells. The major sperm protein released from sperm is detected by receptor proteins on oocytes and gonad cells, which in turn regulates oocyte maturation [22]. However, since *spe-9* mutants, which are reported to have no other defect besides the inability to fertilize eggs, exhibited increased activity, it is possible that embryos are detected by hermaphrodites to regulate locomotor activity. Further research is required to determine the molecular mechanisms underlying the suppression of locomotor activity by the presence of sperm function.

In summary, the results suggest that presence of functional sperm plays an important role in regulating locomotor activity and behavioral states in nematodes. In the absence of functional sperm, hermaphrodites become more active, potentially allowing effective females to encounter more mates. These findings highlight the adaptive significance of regulation of sex-shared behavior by sperm function.
